# Investigation of the “Surgical Cuts CO_2_ Laser Therapy Technique” to Treat Minor Burn Scar Contractures in Children

**DOI:** 10.3390/ebj4030027

**Published:** 2023-07-19

**Authors:** Jennifer Zuccaro, Lisa Lazzarotto, Jamil Lati, Charis Kelly, Joel Fish

**Affiliations:** Division of Plastic and Reconstructive Surgery, The Hospital for Sick Children, Toronto, ON M5G 1X8, Canada

**Keywords:** fractional laser, pediatric, scar, contracture, scar contracture, burn, scar treatment

## Abstract

Fractional carbon dioxide (CO_2_) laser therapy has been shown to improve scar contractures following burns. However, the benefits of using other CO_2_ laser techniques to treat burn scar contractures are relatively unknown. This pilot study investigated a CO_2_ laser technique in which a series of perpendicular “surgical cuts” were created along the contracture. The aim of this study was to evaluate the effectiveness of using the “surgical cuts CO_2_ laser technique” in pediatric patients. This study included 12 participants with minor hand burn scar contractures that received one CO_2_ laser treatment using the surgical cuts technique. Trained assessors measured contractures pre- and post-laser therapy by assessing range of motion (ROM), digit length, and/or hand-span. All contractures were secondary to contact burns with the mean participant age equal to 5.5 years (SD 3.9). For all participants, at least one of the measured characteristics (ROM, hand-span, and digit length) improved after treatment. This pilot study demonstrated the benefit of using the surgical cuts CO_2_ laser technique to treat minor burn scar contractures. Future investigations are needed to further evaluate its effectiveness in comparison to the fractional CO_2_ laser therapy technique.

## 1. Introduction

Despite improvements in the delivery of burn care, many patients continue to develop scar contractures, which can become highly debilitating and negatively impact quality of life. Scar contractures are defined by the abnormal shortening of scar tissue [1]. After a burn injury occurs, the skin undergoes three forms of wound repair including scar formation, re-epithelization, and contraction [2]. During the contraction stage, myofibroblasts contract to help pull the edges of the wound inward leading to its closure. When myofibroblast activity is increased due to an abnormality in the healing process and/or increased tension, a contracture can occur [2,3,4]. Scar contractures that occur over a joint can limit range of motion (ROM) and lead to functional issues. The inability to perform daily activities can be extremely detrimental, particularly to pediatric patients. Palmieri et al. (2012) found that children with hand burn scars who were under the age of five years experienced significantly decreased health-related quality of life and suffered major difficulties with fine and gross motor function [5]. Unfortunately, contractures continue to produce significant morbidity in patients, especially in low-income countries where adequate care is not widely available [6]. Interventions to combat the effect of the strong contractile forces of scarring are critical to prevent permanent loss of function among patients with scar contractures [2].

**Figure 1:**
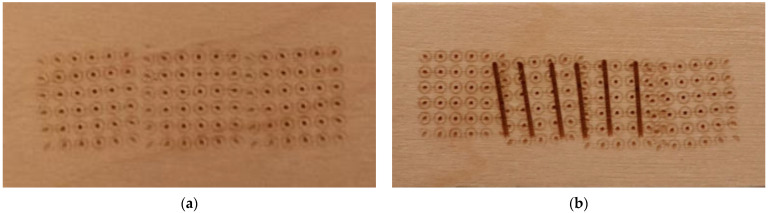
Carbon dioxide laser therapy techniques demonstrated on a tongue depressor. (**a**) Fractional technique; and (**b**) surgical cuts technique.

Before surgical intervention becomes necessary, minor burn scar contractures are often treated with a range of non-operative treatments, such as exercise, massage, splinting, serial casting, pressure garments, silicone preparations, and most recently, carbon dioxide (CO_2_) laser therapy. CO_2_ laser therapy has emerged as a revolutionary treatment for scars and contractures over the last decade. Typically, when CO_2_ laser therapy is used to resurface scar tissue, a fractional technique is applied in which tiny laser microbeams produce deep columns of thermal damage to create a grid-like pattern of holes (Figure 1a) [7,8,9,10]. Previous studies have demonstrated that using a fractional CO_2_ laser technique can soften scar contractures resulting in improvements in ROM and overall function [11,12,13]. However, little is known about the potential benefits of using other CO_2_ laser techniques to treat burn scar contractures. 

Our research group is specifically interested in investigating an alternative CO_2_ laser technique in which the traditional fractional approach is used first and then overlayed with a series of perpendicular “laser surgical cuts” along the length of the contracture (Figure 1b). The idea underlying this technique is that the cuts produced by the laser may help relieve the mechanical tension causing the contracture, thereby leading to improvements in function and cosmesis. Moreover, the technique is non-invasive as the cuts produced by the laser are much smaller and more consistent in terms of depth in comparison to what can be achieved using a scalpel. Our clinical team has used this technique at our institution over the past three years and have documented improvements in both function and cosmetic appearance following treatment. However, to our knowledge, this specific CO_2_ laser technique has not been investigated in a prospective study. To address this knowledge gap, we designed a pilot study to evaluate the effectiveness of using the surgical cuts CO_2_ laser technique to treat minor burn scar contractures in pediatric patients. The primary objective of this study was to determine if using the surgical cuts CO_2_ laser technique is effective in improving the characteristics of minor burn scar contractures in children aged 1 to 18 years.

## 2. Materials and Methods

This prospective, single-arm, interventional pilot study was conducted at a pediatric burn center in Canada that has been verified by the American Burn Association since 2013. At present, our burn center has the largest laser program for pediatric burn scars in Canada and more than 500 laser procedures have been carried out since the technology was implemented in 2014. Institutional ethics approval was obtained prior to commencing this study (protocol code: 100006104) and informed consent was obtained from all participants/caregivers. Eligible participants were recruited from the outpatient burn clinic and included those who: (1) were aged 1–18 years at presentation; and (2) had a minor burn scar contracture that crossed a joint (defined as a contracture where there has been a loss of <30% of the ROM of the affected joint). Exclusion criteria included: (1) patients with a moderate or severe burn scar contracture (defined as a contracture where there has been a loss of ≥30% of the ROM of the joint); and (2) patients with a history of keloid scarring.

All outcome measurements were performed by trained occupational and/or physiotherapists at the following timepoints: (1) immediately before CO_2_ laser therapy was administered (i.e., on the same day as the procedure), and (2) at the first clinic visit following the procedure (approximately one-week post-laser treatment). Depending on the contracture’s location and extent of involvement, the trained therapist(s) examined at least one of the following characteristics at each study visit: (1) ROM: measured by placing a metal goniometer on the dorsum of the affected finger with the center of the tool aligned at the joint lacking full ROM; (2) digit length: measured by placing a straight ruler on the palmar side of the hand, from directly below the affected finger on the distal palmar crease to the tip of the finger; and (3) hand-span: measured with a straight ruler from the tip of the little finger to the tip of the thumb while the hand was passively stretched. The pre- and post-laser therapy measures were compared, and the Wilcoxon test was used to analyze the available data.

**Figure 2:**
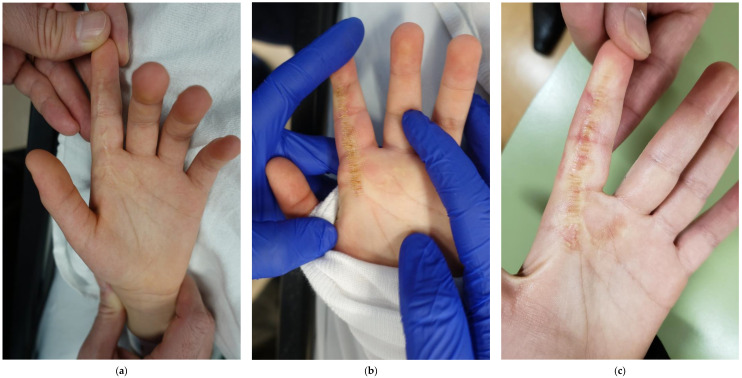
Visual depiction of surgical cuts treatment algorithm. (**a**) Minor burn scar contracture on second digit of left hand; (**b**) depiction of surgical cuts immediately following laser treatment; (**c**) one week following surgical cuts immediately after removal of the plaster splint.

All CO_2_ laser treatments were performed using a 10,600 nm CO_2_ laser device (Syneron Candela CO2RE, Wayland, MA, USA) using the standardized treatment algorithm below (Figure 2):

1. Identify the discrete, linear band causing the minor scar contracture.
2. Visually examine and manually palpate the contracture to determine the perceived thickness and adjust laser energy settings accordingly (typical settings for “fusion mode” include core energy = 70 mJ, ring energy = 48–55.2 mJ, and fractional coverage = 20–25%).
3. Use the traditional, fractional CO_2_ laser setting to create a grid-like pattern of holes along the length of the contracture and apply the same technique to any adjacent scarring surrounding the contracture.
4. Adjust the CO_2_ laser settings to the “surgical mode” to create a series of linear cuts that are perpendicular to the contracture.
5. Determine the appropriate width of the laser beam by measuring the visible width of the contracture and extending it by 2 mm to ensure appropriate coverage. When using the surgical cuts technique, initial laser energy settings should not exceed 25 mJ to ensure patient safety. The treating clinician may try a test pulse and then gradually increase the energy settings until the desired effect is achieved.
6. Space surgical cuts as close together as is feasible without overlapping.
7. Apply topical triamcinolone (40 mg/mL) and topical xylocaine to the laser-treated area. Place digits and hand in a stretched position and place in a plaster splint (not circumferential) for one week. Following removal, commence stretching exercises and/or rehabilitation therapy and/or night splinting and return to daily activities.

As per our routine practices, all laser procedures were performed in the operating room or in a satellite anesthesia room under intravenous sedation (fentanyl ± morphine ± adjuvant(s)) to minimize pain and ensure treatment accuracy [14].

## 3. Results

Twelve pediatric patients with a minor scar contracture resulting from a burn injury to the hand participated in this study (Table 1). The mean participant age at the laser procedure was 5.5 years (SD 3.9). All burns resulted from contact injuries such as touching a hot object (e.g., a stove or light source) or falling into hot coals from a campfire. Contractures of various durations were treated; time since the initial burn injury to the laser procedure ranged from 2 months to 174 months. As demonstrated in Table 2, improvements in ROM, hand-span, and/or digit length were identified in all patients at the follow-up visit approximately one week following the laser procedure. More specifically, statistically significant improvements in digit length and ROM were found (*p* < 0.05). No complications were reported either during the procedure or at the follow-up visit.

## 4. Discussion

The positive results observed in this preliminary study warrant the consideration of the surgical cuts CO_2_ laser therapy technique for patients with minor burn scar contractures. In all cases, at least one of the measured contracture characteristics including ROM, hand-span, and/or digit length improved after a single CO_2_ laser treatment. These findings are particularly pertinent as they highlight the potential role of laser therapy as a method to accelerate contracture improvement and possibly avoid more invasive future interventions such as reconstructive surgery.

Over the past decade, CO_2_ laser therapy has become a widely accepted treatment for scars and contractures with reported improvements in scar thickness, stiffness, and overall appearance [15,16,17,18]. However, most applications of CO_2_ laser therapy for scars involve using a fractional technique in which a grid-like pattern of holes is ablated to facilitate collagen remodelling and improve scar outcome [19,20,21]. Although the precise molecular mechanisms that underlie these improvements have not been fully elucidated, it is thought that columns of collagen are removed via ablation while the surrounding uninjured tissue serves as a reservoir to facilitate collagen remodelling and neocollagenesis [7,9,10,19,20,21,22,23,24]. Whilst many studies have detailed the fractional technique for resurfacing symptomatic scars, research regarding its utility in improving contractures is limited, as few contracture-specific studies have been published to our knowledge [11,12,13]. Despite the limited body of evidence, the available studies have demonstrated improvements in contracture characteristics such as ROM and functionality, thereby supporting the role of CO_2_ laser therapy in scar contracture treatment paradigms [11,12,13].

Fractional CO_2_ laser therapy is not presently considered a gold standard treatment for scar contractures. As such, our research group began examining various CO_2_ laser settings for the treatment of scar contractures, which led us to investigate the surgical cuts laser technique in the present study. As described in our treatment algorithm, the surgical cuts technique involves creating a series of lines along the length of the contracture. Although we have not yet established the mechanism of action, we hypothesize that the surgical cuts technique allows for the physical disruption of the contracture, thereby allowing the release of mechanical tension. We believe that this reduction in tension is responsible for the rapid improvements in contracture characteristics observed in our study. Several advantages that we have observed in our clinical experience using the surgical cuts technique include: (1) immediate improvements following one treatment session; (2) easier recovery than a surgical scar release such as a z-plasty; and (3) the ability to repeat the procedure if necessary (e.g., patient undergoes a growth spurt).

A key aspect of our laser treatment approach for contractures is to institute the surgical cuts laser technique when standard non-operative therapy has been deemed unsuccessful by the clinical team (e.g., no improvement or worsening of the contracture). Although this is not always possible in patients with late referrals (e.g., patients who are referred many years after their initial injury), patients who receive acute care at our institution are closely followed by rehabilitation therapists and are typically offered laser therapy early on. In our experience, early laser treatment has not been associated with increased pain or complications. We concur with the idea proposed by Shumaker et al. (2012) that implementing laser therapy during the active remodelling phase of wound healing could positively impact the overall trajectory of the scar [12]. In particular, early laser intervention may enable contractures to respond better to subsequent treatment thereby reducing: (1) the need for future surgery; or (2) the complexity of future surgical procedures [14,25,26]. This potential shift in surgical care could impact overall healthcare costs with a reduction of resource use related to operating time, in-patient admissions, and rehabilitation [26]. It is also worth noting that the pediatric population stands to gain particular benefit from this early treatment approach as the development or worsening of contractures can impede function and overall growth. Despite the potential advantages of the early treatment approach, future studies are needed to provide rigorous long-term data that evaluate patients at predetermined time points (e.g., 6 months, 1 year, and 2 years).

Although the surgical cuts technique is non-invasive, the impact on patients and their families should be considered when comparing it to other non-operative approaches. More specifically, patients must wear a plaster splint for one week as opposed to a light dressing for two days (as would be typical practice for fractional CO_2_ laser therapy). Also, an additional visit to the hospital one-week post-laser for removal of the splint and a review of exercises and/or additional splint fabrication may inconvenience both the patient and their caregiver.

Given that this was a pilot study, there are several limitations aside from the small sample size that must be taken into consideration. Firstly, because a control group (either standard fractional CO_2_ laser therapy alone or conservative treatment alone) was not included in the present study, future studies are needed to confirm which technique is superior. Secondly, because the surgical cuts CO_2_ laser technique was used in combination with topical triamcinolone and post-laser plaster splinting, we cannot conclude if the therapies worked synergistically or if a single aspect of the treatment algorithm was most responsible for the improvements. Thirdly, although the trained assessors were blinded to the measurements obtained prior to initiating treatment, they were aware that each participant received treatment with a new laser technique, which may have introduced the potential for rater bias. In future studies, independent assessors who are unaware of each participant’s treatment plan will be included in the study design. Fourthly, the laser settings used for each participant were selected by the laser operator based on their clinical evaluation (through observation and manual palpation) rather than an objective measurement of each contracture’s characteristics. Whilst we are not proposing to standardize the laser settings to utilize a “one size fits all approach”, it would be advantageous to incorporate objective measurements such as ultrasound [27,28], or optical coherence tomography [29], to help guide laser settings. While our study evaluated early outcomes, a long-term follow-up is needed to evaluate if the improvements observed immediately following laser treatment are sustained over time. Future long-term studies are needed to address this question as well as the impact that the surgical cuts CO_2_ laser technique has on the need for future surgical revisions.

## 5. Conclusions

The application of laser therapy for the treatment of scars and contractures is vast with numerous lasers available, each with their own adjustable settings. Although CO_2_ laser therapy has been widely utilized in scar-focused studies, most research is based on using the traditional, fractional technique. Our study demonstrated the clinical innovation of using the CO_2_ laser to produce a series of surgical cuts to improve scar contractures. To our knowledge, this is the first study to detail the surgical cuts CO_2_ laser therapy technique for treating minor burn scar contractures and our findings demonstrate that this novel technique can produce improvements in scar characteristics including ROM, hand-span, and digit length. A larger investigation with long-term follow-up is needed to determine if the surgical cuts technique is more effective than using traditional, fractional CO_2_ laser settings and if the long-term benefits can be maintained following treatment.

## Figures and Tables

**Table 1:** Patient demographics.

Gender	*n* (%)
Male	5 (41.7)
Female	7 (58.3)
Participant Age at Laser Treatment	*n* (SD)
Mean (SD)	5.5 years (3.9)
Range	1–15 years
Fitzpatrick Skin Type	*n* (%)
I	4 (33.3)
II	5 (41.7)
III	1 (8.3)
IV	2 (16.7)
Burn Etiology	(*n*, %)
Contact	12 (100)
Burn Depth	(*n*, %)
Partial thickness	2 (16.7)
Full thickness	5 (41.7)
Undocumented due to acute treatment at different institution	5 (41.7)
Acute Burn Treatment	(*n*, %)
Conservative therapy	3 (25.0)
Skin grafting	4 (33.3)
Undocumented due to acute treatment at different institution	5 (41.7)
Age of Scar at Laser Treatment	(*n*, SD)
Mean (SD)	39.7 months (52.0)
Range	2–174 months

**Table 2:** Pre- and post-laser therapy measurements.

ID	Location of Affected Joint(s)	Pre-Laser Range of Motion	Post-Laser Range of Motion	Pre-Laser Digit Length	Post-Laser Digit Length	Pre-Laser Hand- Span	Post-Laser Hand- Span
1	Right D ^†^ 5 PIP ^§^ Right D5 DIP ^‡^	Passive −55° extension Passive −50° extension	Passive 0° (full extension) Passive −10° extension				
2	Right D5 PIP	Passive −15° extension	Passive −10° extension			12 cm	13 cm
3	Left D2 PIP	Active −30° extension	Active 0° (full extension)	7.9 cm	8.6 cm		
4	Left D3 Left D4			5.2 cm 5.0 cm	5.3 cm 5.7 cm	15.5 cm	15.5 cm
5	Right D3 Right D4			3.5 cm 4.0 cm	4.5 cm 4.0 cm		
6	Right D2 DIP	Active −25° extension	Active −15° extension				
7	Right D4 DIP	Active −15° extension	Active 0° (full extension)	6.5 cm	6.9 cm		
8	Left D1 IP *	Active −60° Extension	Active 0° (full extension)				
9	Right D2 DIP	Active −30° extension	Active 0° (full extension)	5.5 cm	6.0 cm		
10	Right D4 DIP	Passive −15° extension	Passive 0° (full extension)				
11	Left D5 PIP	Passive −5° extension	Passive 0° (full extension)	8.5 cm	8.5 cm		
12	Left D5 DIP	Passive −15° extension	Passive 0° (full extension)				

^†^ “D” refers to the affected digit; ^§^ “PIP” refers to the proximal interphalangeal joint; ^‡^ “DIP” refers to the distal interphalangeal joint; * “IP” refers to the interphalangeal joint.

## Data Availability

The data presented in this study are available on request from the corresponding author. The data are not publicly available due to privacy restrictions.
